# Atypical antipsychotic augmentation in SSRI treatment refractory obsessive-compulsive disorder: a systematic review and meta-analysis

**DOI:** 10.1186/s12888-014-0317-5

**Published:** 2014-11-29

**Authors:** David Veale, Sarah Miles, Nicola Smallcombe, Haben Ghezai, Ben Goldacre, John Hodsoll

**Affiliations:** The Institute of Psychiatry, King’s College London and South London and Maudsley NHS Foundation Trust, 16 De Crespigny Park, Denmark Hill, London, SE5 8AF UK; Kings College London Medical School, Denmark Hill, London, SE5 9RS UK; London School of Hygiene & Tropical Medicine, Keppel Street, London, WC1E 7HT UK; Centre for Anxiety Disorders and Trauma, The Maudsley Hospital, 99 Denmark Hill, London, SE5 8AZ UK

**Keywords:** Obsessive compulsive disorder, Anti-psychotic, Selective serotonergic reuptake inhibitor

## Abstract

**Background:**

In 2006, the National Institute of Clinical and Health Excellence (NICE) guidelines for Obsessive Compulsive Disorder (OCD) recommended anti-psychotics as a class for SSRI treatment resistant OCD. The article aims to systematically review and conduct a meta-analysis on the clinical effectiveness of atypical anti-psychotics augmenting an SSRI.

**Methods:**

Studies that were double-blind randomized controlled trials of an atypical antipsychotic against a placebo, for a minimum of 4 weeks, in adults with OCD, were included. Yale-Brown Obsessive Compulsive Scale (Y-BOCS) scores were the primary outcome measure. Inclusion criteria included Y-BOCS score of 16 or more and at least one adequate trial of a SSRI or clomipramine for at least 8 weeks prior to randomization. Data sources included Medline, Embase, PsycINFO, Cochrane Database of Systematic Reviews (CDSR), trial registries and pharmaceutical databases and manufacturers up to September 2013. Forest-plots were drawn to display differences between drug and placebo on the Y-BOCS.

**Results:**

Two studies found aripiprazole to be effective in the short-term. There was a small effect-size for risperidone or anti-psychotics in general in the short-term. We found no evidence for the effectiveness of quetiapine or olanzapine in comparison to placebo.

**Conclusions:**

Risperidone and aripiprazole can be used cautiously at a low dose as an augmentation agent in non-responders to SSRIs and CBT but should be monitored at 4 weeks to determine efficacy.

## Background

The National Institute of Clinical and Health Excellence (NICE) Guidelines for Obsessive Compulsive Disorder (OCD) in 2006 [1] recommended that for adults with OCD, with no response to a full trial of at least one Selective Serotonin Reuptake Inhibitor (SSRI) or clomipramine alone, and a full trial of combined treatment with Cognitive Behaviour Therapy (CBT) – that includes exposure and response prevention (ERP) as well as an SSRI, the following treatment options should be considered: (1) Additional CBT of increased intensity, (2) Adding an antipsychotic to an SSRI or clomipramine, (3) Combining clomipramine and citalopram. No guidance was given on the order of options. Antipsychotic drugs were recommended as a class and no advice was provided on *how* to use an antipsychotic e.g. the dose, duration or potential risk in the long-term. The recommendation was based on a meta-analysis of 5 randomized controlled trials (RCTs) of haloperidol (1 double-blind), risperidone (2 double-blind), quetiapine (1 single-blind) and olanzapine (1 double-blind) compared with a placebo and various open-label studies. The same guidelines did not recommend adding an antipsychotic to a SSRI for people with Body Dysmorphic Disorder (BDD) on the basis of one negative trial with pimozide in those resistant to a SSRI [2]. The NICE Evidence Update [3] (which summarized the evidence published since the NICE guidelines from 2005 to 2013) included a Cochrane review in 2010 [4] of a meta-analysis of 11 atypical antipsychotics in OCD, analyzing only a categorical measure of recovery. Since then, further RCTs of antipsychotics have been published. Current reviews continue to recommend antipsychotic drugs as a class for augmentation of SSRI treatment resistant OCD [5,6]. It was considered timely to conduct a new systematic review and meta-analysis given the potential long-term risks of antipsychotics. Furthermore, off-label prescribing may be particularly vulnerable to selective data publication, particularly since trials of such uses have been specifically exempted from industry pledges on transparency: this can lead to exaggeration of treatment benefits [7]. A systematic search for unpublished studies was therefore also planned for inclusion. Our question for the systematic review was: “For adults who have OCD which has failed to respond to at least one trial of a serotonergic reuptake inhibitor, will an antipsychotic drug be more effective than a placebo, in reducing obsessive-compulsive symptoms?” Our secondary aim was to determine if guidance could be provided for the order of stepped care, and for the dose and duration for a trial of an anti-psychotic.

## Methods

The review aimed to include any double blind randomized study that investigated the effects of an atypical antipsychotic compared with a placebo for adults with OCD and which used an intention-to-treat analysis. The PRISMA method of reporting was used [8].

We excluded haloperidol from the review as, although it was included in the original meta-analysis by NICE, there is only one early RCT [9], and of all the antipsychotics it is the most likely to cause extra-pyramidal side effects or be discontinued for any reason [10]. We focused on the potential benefits of atypical antipsychotics as potential harms in the long-term are well documented in other populations (for example weight gain, metabolic syndrome, extra-pyramidal symptoms, sedation) [10] and no long term studies have been conducted in OCD [4].

### Eligibility criteria

Studies were included if:

- (1) They described adults who had a diagnosis of OCD according to the DSM or ICD.
- (2) They used the Yale-Brown Obsessive Compulsive Scale (Y-BOCS) [11] as a primary outcome measure. The Y-BOCS is a 10-item clinician-rated scale which is widely used to measure the severity of obsessive-compulsive symptoms, which has a total score range of 0 to 40. Higher scores indicate greater symptomatology of OCD.
- (3) Participants had persistent symptoms of OCD defined as a Y-BOCS score of 16 or more.
- (4) Participants had had at least one adequate trial of a SSRI or clomipramine. An adequate trial of a SSRI or clomipramine was defined as a maximum dose tolerated for at least 8 weeks prior to randomization.
- (5) Participants remained on the SSRI or clomipramine for the duration of the trial.
- (6) They had a trial end point of at least 4 weeks.

No publication date or publication status restrictions were imposed.

### Information sources

Medline, Embase, PsycINFO and Cochrane Database of Systematic Reviews (CDSR), clinical trial registries and pharmaceutical databases up to December 2013 were used to obtain published and unpublished data.

### Search

The Medline search strategy used for the NICE guidelines was translated into comparable search strategies for Embase and for previous systematic reviews in the Cochrane Database (CDSR). We searched all international clinical trial registries and databases of the pharmaceutical manufacturers and wrote to the manufacturers to enquire about any unpublished data of any antipsychotic used in OCD.

### Study selection

A full-text article was retrieved for any citation deemed relevant by any of the reviewers. All full text articles were reviewed for inclusion by at least two of the authors. Studies were selected if they fulfilled the eligibility criteria.

### Data collection process

Information was extracted from each included trial on: (1) The number of participants in each intervention group (2) Mean *(M)* and standard deviation (*SD*) Y-BOCS scores measured at pre and post drug and placebo intervention in order to generate related Forest plots.

- Two studies provided incomplete data:
- (a) For McDougle et al., [12] we calculated the mean and standard deviation from the raw data provided and used Last Observation Carried Forward (LOCF) methodology for three participants whose data were missing post observation (risperidone *M* = 19.45, *SD* = 8.19 and placebo *M* = 25.43, *SD* = 4.58). We compared the estimates of treatment effect given using LOCF, to effects calculated after excluding data from drop-outs. Estimates of treatment effect did not differ across the methods, therefore LOCF was used for the final analysis to ensure intention-to-treat analysis was used, and that drop-outs were not assumed to behave in the same way as completers, therefore reducing bias.
- (b) Shapira et al., [13] was contacted who provided the week 8 data for the olanzapine group (*M* = 19.27, *SD* = 3.40) and placebo *M* = 9.64 *SD* = 4.14.

We either extracted from all the studies or wrote to the corresponding author for the (1) Dose range of the antipsychotic (mg/day), (2) Trial duration of antipsychotic (in weeks), (3) Current SSRI minimum duration (in weeks) before randomization and whether participants had received the SSRI as part of a double-blind or open-label trial or part of routine care before commencing the antipsychotic drug, (4) Number of previous SSRI trials received by participants before recruitment, (5) Number of previous CBT trials received before recruitment, (5) Inclusion criteria on the Y-BOCS, (6) SSRI treatment resistant description (see Table 1).

**Table 1:** **All studies of SSRIs augmented by atypical anti-psychotic in OCD with their characteristics**

**Study**	**Drug (** ***n*** **)**	**Dose [** ***mg/d*** **]**	**Trial duration (** ***weeks*** **)**	**Current SSRI min. length (** ***weeks*** **)**	**Previous SSRIs prior to recruitment**	**Previous CBT**	**SSRI treatment resistant description**
	**Placebo (** ***n*** **)**	**(** ***M, SD*** **)**					
McDougle, 2000 [12]	Risperidone (20)	1 - 6	6	12 (open label of a SSRI or clomipramine)	58.3% had at least 2 trials of SRIs	30% at least 1 trial	≤ 35% improvement or Y-BOCS ≥16 and no better than score of 3 (minimal improvement) on CGI to SSRI
	Placebo (16)	(2.2, 0.7)					
Hollander, 2003 [14]	Risperidone (10)	0.5 - 3	8	12 (routine care SSRI)	100% had at least 2 trials SRIs	62.5% at least 1 trial	No better than score of 3 (minimal improvement) on CGI to SSRI. No minimum severity on Y-BOCS specified
	Placebo (6)	(2.25, 0.86)					
Erzegovesi, 2005 [15]	Risperidone (10)	0.5	6	12 (open label fluvoxamine)	100% had at least 1 trial of a SRI	None	35% or greater improvement or Y-BOCS ≥16 and no better than score of 3 (minimal improvement) on CGI to SSRI
	Placebo (10)	(fixed dose)					
Simpson, 2013 [16]	Risperidone (20)	0.5 – 4	8	12 (routine care SSRI)	80% had at least 2 trials of SRIs	7% at least 1 trial	All but two had at least minimal improvement to a SSRI (i.e. some partial responders) and Y-BOCS ≥16
	Placebo (40)						
	CBT (40)						
Storch, 2013 [17]	Paliperidone (17)	3 - 9	8	12 (routine care SSRI)	100% had at least 2 trials	0% had at least 1 trial	Not formally assessed but “SSRI had had minimal effect”. Y-BOCS ≥19
	Placebo (17)						
Bystritsky, 2004 [18]	Olanzapine (13)	5 - 20	6	12 (routine care, SSRI)	100% had at least 2 trials	100% at least 1 trial	No specific criteria
	Placebo (13)	(11.2, 6.5)					
Shapira, 2004 [13]	Olanzapine (22)	5 - 10	6	8	40.9% had at least 1 trial of SRI	*Not known*	<25% improvement and score of 4 (moderate) or greater on CGI and Y-BOCS ≥16
	Placebo (22)	(6.1, 2.1)		(open label fluoxetine)			
Denys, 2004 [19]	Quetiapine (20)	200	8	8 (routine care SSRI)	100% had 2 or more trials	72.5% at least one trial	< 25% improvement on Y-BOCS to SSRI and Y-BOCS ≥18
	Placebo (20)	(*Range* 100–300)					
Carey, 2005 [20]	Quetiapine (20)	25 - 300	6	12 (routine care SSRI)	*Not known*	*Not known*	< 25% improvement on Y-BOCS or no better than score of 3 (minimal improvement) on CGI to SSRI. No minimum Y-BOCS specified
	Placebo (21)	(168.75, 120.82)					
Fineberg, 2005 [21]	Quetiapine (11)	25 - 400	16	12 (routine care SSRI)	*Not known*	*Not known*	< 25% improvement on Y-BOCS to SSRI and Y-BOCS ≥ 18
	Placebo (10)	(215, 124)					
Kordon, 2008 [22]	Quetiapine (20)	400 - 600	12	12 (routine care SSRI)	17.5% had two or more trials 82.5% had at least one trial	100% at least one trial	< 25% improvement on Y-BOCS to SSRI and Y-BOCS ≥18
	Placebo (20)						
Diniz, 2011 [23]	Quetiapine (18)	50 - 200	12	8 (open label fluoxetine)	Most had failed their first adequate SSRI trial	11% at least 1 trial	<35% improvement on Y-BOCS to SSRI and Y-BOCS ≥16
	Placebo (18)	(142, 65)					
	Clomipramine (18)						
Muscatello, 2011 [24]	Aripiprazole (20)	15	16	12 (routine care SSRI)	*Not known*	*Not known*	Y-BOCS ≥16
	Placebo (20)	(fixed dose)					
Sayyah, 2012 [25]	Aripiprazole (21)	10	12	12 (routine care SSRI)	*Not known*	*Not known*	Y-BOCS ≥21
	Placebo (18)	(fixed dose)					

### Data item

Our primary outcome of interest was the change in Y-BOCS score. We calculated difference in means (pre and post) for each group and entered change-from-baseline standard deviations calculated by following Cochrane guidelines on how to impute missing standard deviations. It was possible to calculate *r* from the statistics output of two of the papers included in the meta-analysis [19,22]. The mean of these two *r* values was then taken, and gave *r* = 0.4. Sensitivity analyses testing *r* = 0.2 and *r* = 0.6 indicated that as *r* increased or decreased, the significance of tests remained the same. Therefore, our Pearson’s *r* value from which to compute the change standard deviation was kept at 0.4, based on previous research. This gave us 3 key variables for each group (drug/placebo), necessary for computing a meta-analysis and analyzing the effect-sizes of study outcomes; *n* in group, mean difference in Y-BOCS outcome score over the intervention duration, related change-from-baseline standard deviation. If the mean Y-BOCS or other information was not available we contacted the corresponding author of the paper.

All the studies used different definitions of recovery of either >25% [14,16] or >35% [12,15,17] reduction in Y-BOCS outcome either with or without additional criteria such as change on Clinical Global Improvement scale [12,15]. We therefore confined our categorical analysis to risperidone studies only since the previous Cochrane review [4] had conducted this analysis on other anti-psychotics and there were no new studies for quietapine and olanzapine.

### Risk of bias in individual studies

We assessed the risk of bias at a study level using the GRADE system [26], which is a systematic and explicit approach to making judgements about quality of evidence and strength of recommendations.

### Summary measure

Our principal summary measures were the difference in means in the Y-BOCS score from pre to post, and the related effect-size (Cohen’s *D*).

#### Synthesis of results

**Figure 1:**
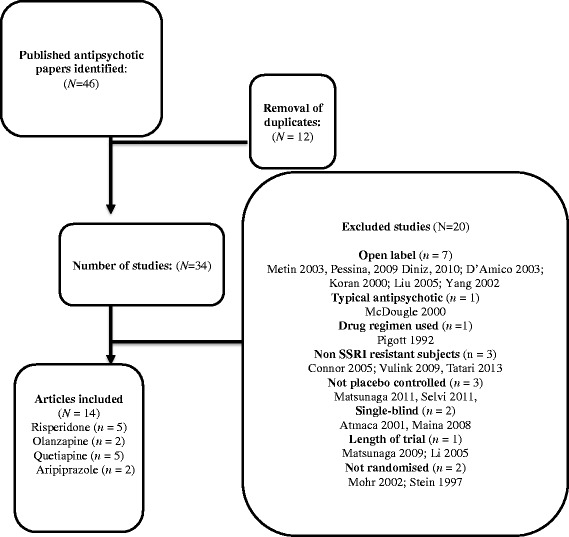
**Meta-analysis of all anti-psychotics for obsessive-compulsive disorder.**

**Figure 2:**
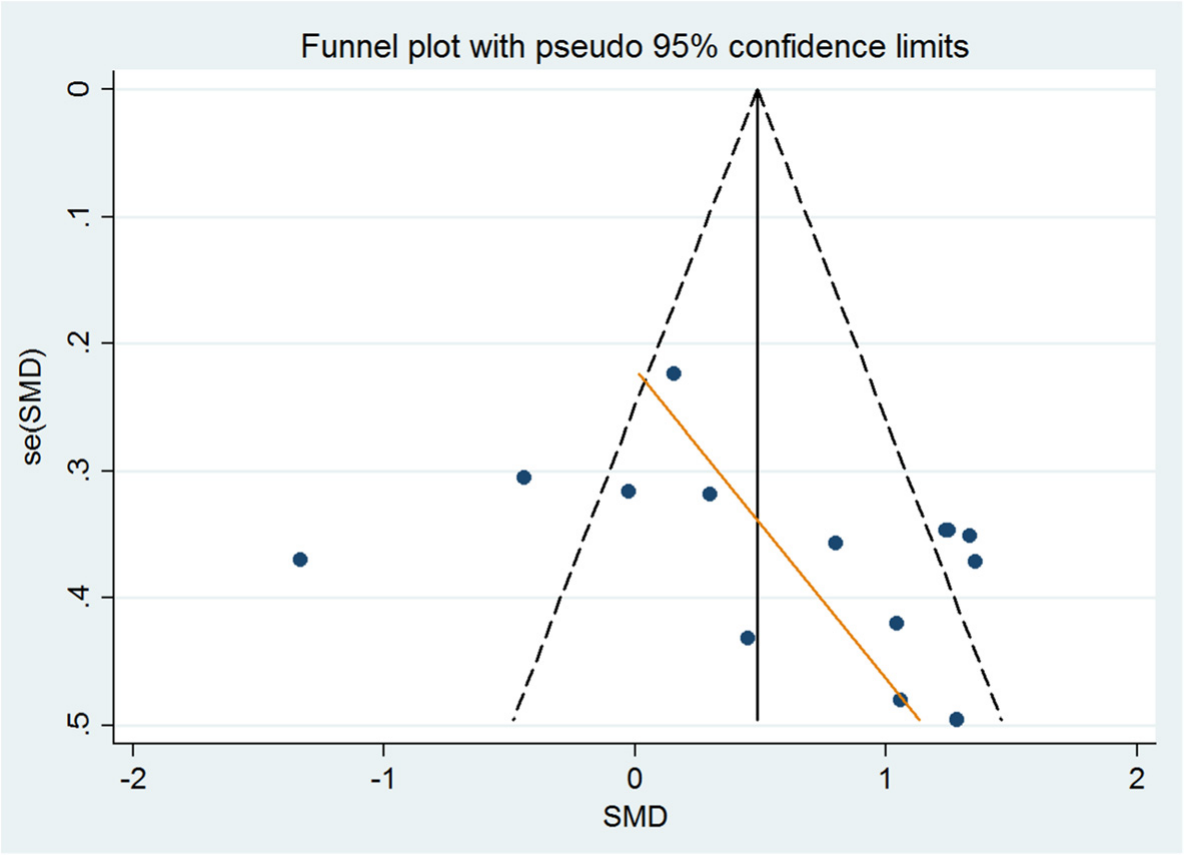
**Meta-analysis of risperidone treatment vs placebo for obsessive-compulsive disorder.**

**Figure 3:**
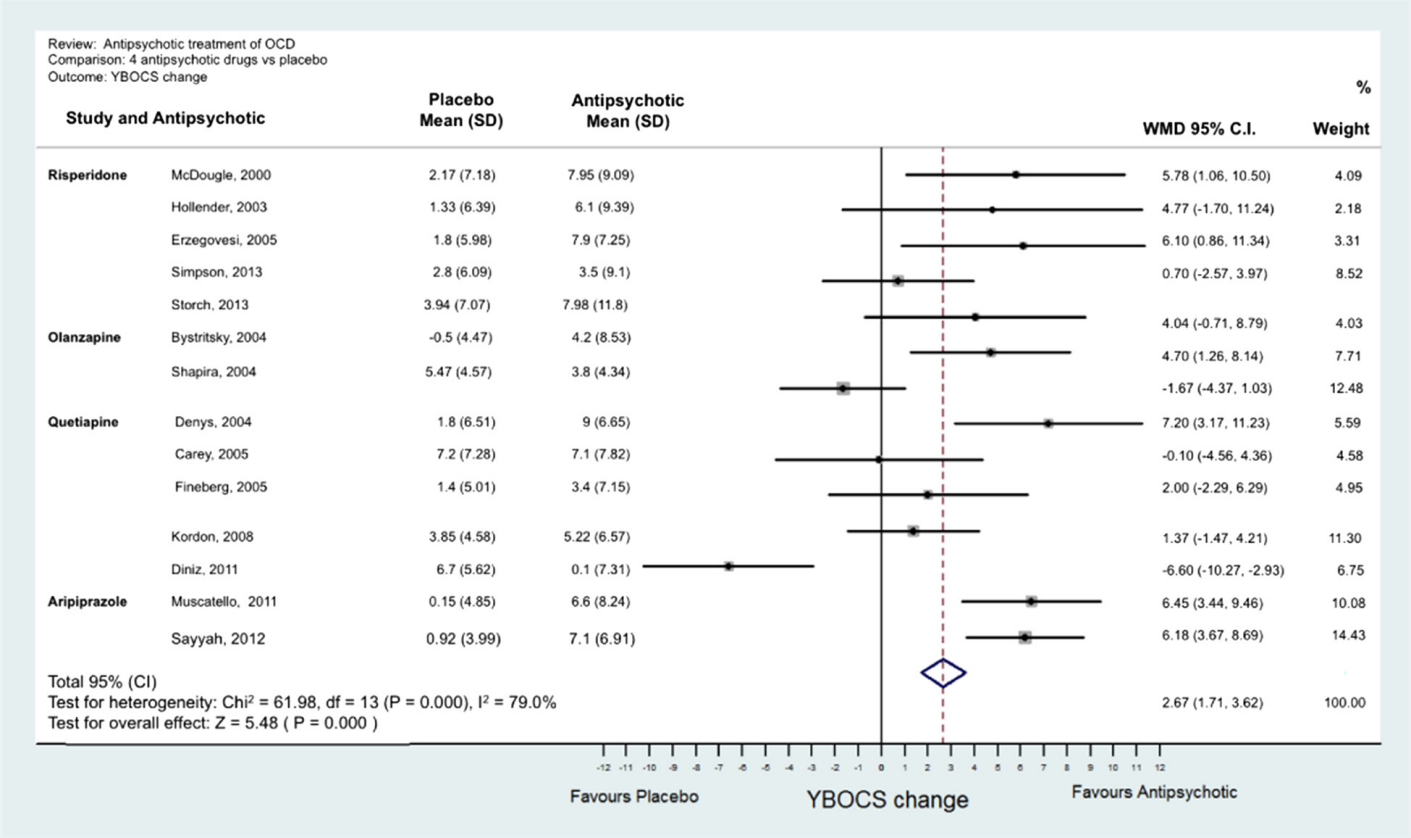
**Meta-analysis of olanzapine treatment vs placebo for obsessive-compulsive disorder.**

**Figure 4:**
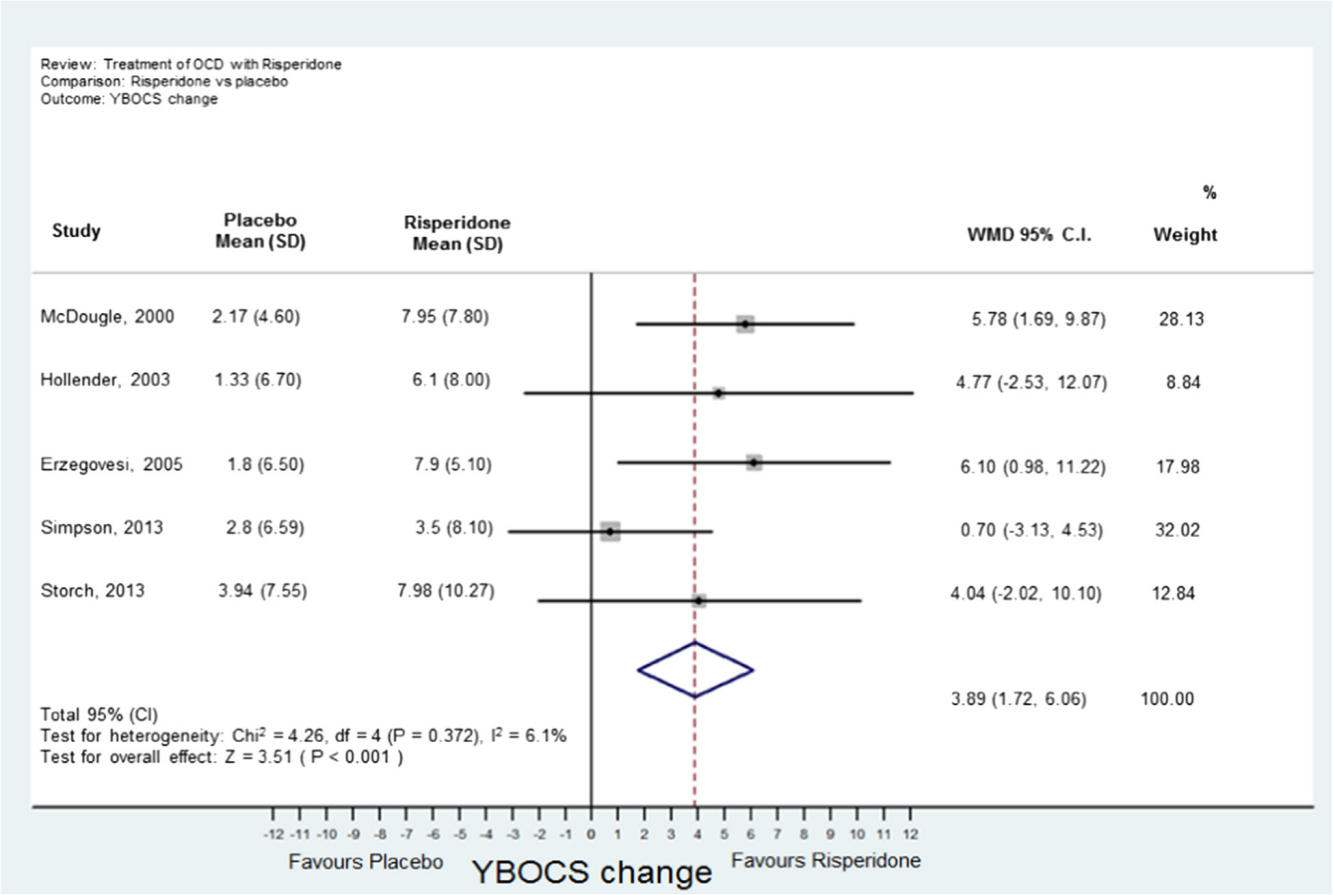
**Meta-analysis of quetiapine treatment vs placebo for obsessive-compulsive disorder.**

**Figure 5:**
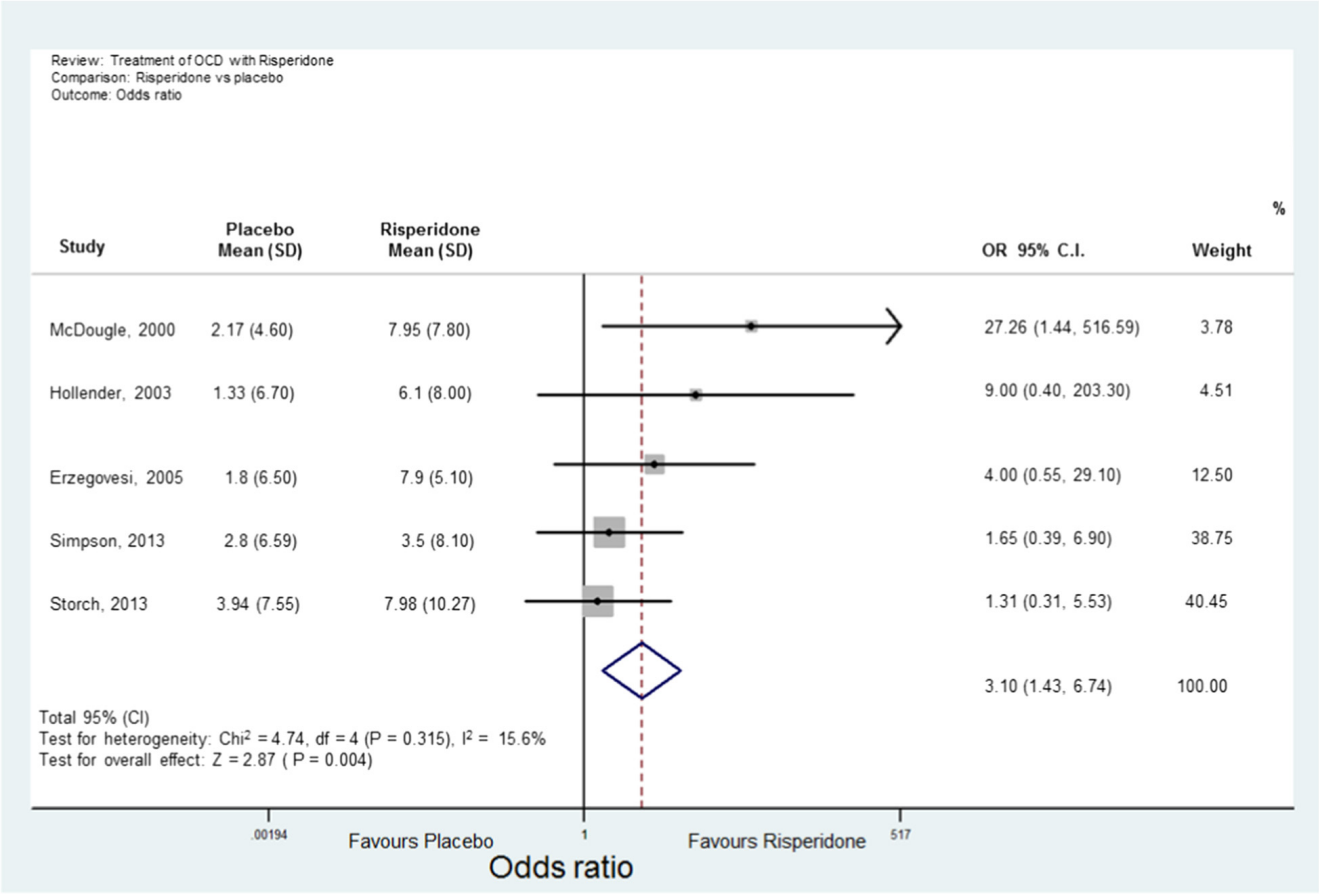
**Meta-analysis of aripiprazole treatment vs placebo for obsessive-compulsive disorder.**

Analyses were conducted using “metan” and associated commands in STATA, version 11 [27,28]. The command combined the outcome of each drug to give an overall difference on the original Y-BOCS scale (shown along the x-axis of Figures 1, 2, 3, 4 and 5), summary effect-size and 95% confidence intervals (*C.I.*), using a random effects meta-analysis model of continuous data, with each study change score weighted by the inverse of the variance [29]. We used a random effects model as we assumed that the included studies are a random sample of the population of studies. Forest plots (see Figures 1–5) were created – each line depicting estimates and confidence intervals for each study, and plotting symbol size representing the weight of each study entered into the meta-analysis. Further, as the clinical populations and treatments had varying factors, we expected treatment effects to be heterogeneous. For each Forest plot of studies separated according to antipsychotic trialed, a Z-score was computed to demonstrate the significance of the overall effect of a drug in comparison to a placebo. For investigating the effects of risperidone, a categorical meta-analysis was also conducted to determine the odds ratio for responding in comparison to not responding on the Y-BOCS. One of the studies found 0 responders in the placebo arm [12]. STATA performs poorly for studies with a very low or very high event rate and so by default changes zero frequencies to 0.5 in order to give a minimum variance unbiased estimate. As this procedure can influence weighted mean differences, the categorical analysis has been included for research comparison purposes but is to be considered with caution.

### Risk of bias across studies

Heterogeneity across studies was assessed visually with a Forest plot and statistically with the Q statistic (21) and I2 statistic. Asymmetry and publication bias of the data was assessed by a Funnel Plot. However the small number of studies and participants for each individual drug made it difficult to interpret [30].

## Results

### Study selection

**Figure 6:**
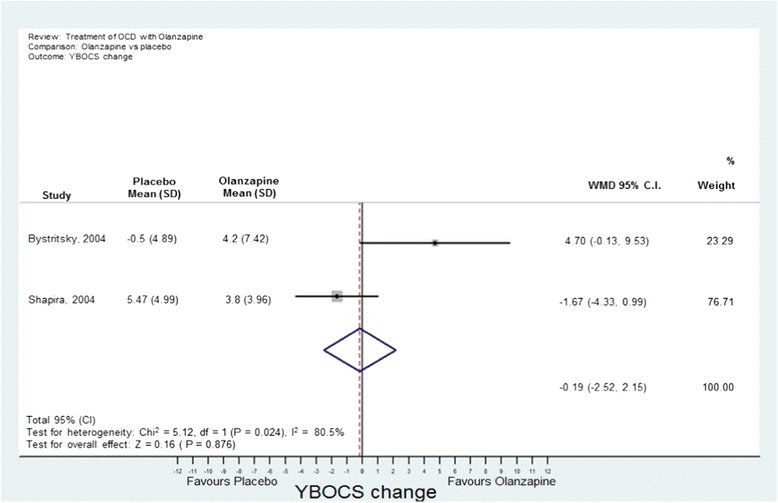
**Flow diagram of study selection for meta-analysis.**

Figure 6 provides a flowchart of the search and the number of studies that were screened for eligibility and subsequently excluded or included in the review. Our search of trial registries found one published study of risperidone or placebo with a SSRI in a non-indexed journal which showed no benefit from adding risperidone [31]. However this did not meet our inclusion criteria as participants were not resistant to a SSRI. No unpublished studies were found from trial registries or received from manufacturers.

### Study characteristics

The characteristics of all studies extracted for inclusion in the meta-analysis are shown in Table 1.

### Risk of bias across the studies

**Figure 7:**
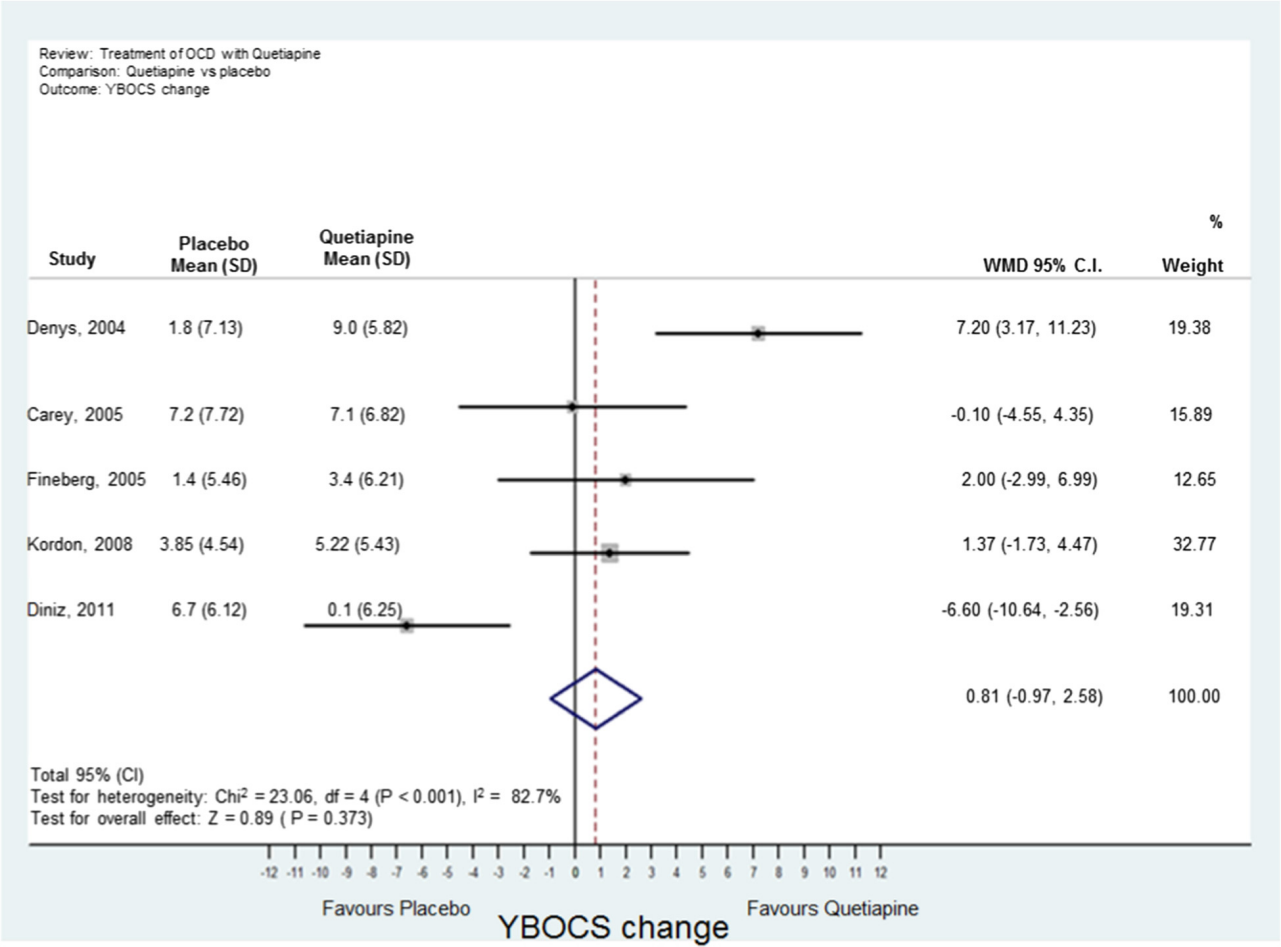
**Funnel plot for all studies.**

A Funnel plot for all the studies was drawn (Figure 7). There is some suggestion of asymmetry in the funnel plot, however as all studies included in the analysis were small it is difficult to draw a firm conclusion in terms of small study bias. Asymmetries in funnel plots can also be due heterogeneity within the sample and over-estimation of treatment in some studies. Given this, we would advise caution in any conclusion of publication bias.

For the GRADE system [26], 4 points was awarded, as they were all RCTs.

The Quality dimension was rated as “-2”. All of the trials had a small sample size. There were no long-term follow up data. Most studies used intention-to-treat analysis with last observation carried forward (LOCF) for missing data. However LOCF carries a risk of bias and variance of treatment effect will be under-estimated as natural variation in measurement is factored out [32]. One study used intention to treat analysis as well as hot-deck imputation [23], and another used multiple imputation [17] to amend for missing data. Only multiple imputation is recommended as statistically unbiased way of dealing with missing data. None of the trials had any self-report outcome measures of obsessive-compulsive symptoms or quality of life thus making the conclusions less safe as blindness may have been compromised.

Consistency was rated as “0”. The I^2^ and Q values indicate that there was significant heterogeneity between the olanzapine trials and the quetiapine trials. However the small number of trials means that the estimate may not be reliable.

Directness was rated as “-1”. There were different inclusion criteria and dosing of drugs. The populations recruited were narrow in terms of not recruiting those that failed CBT.

Effect size was rated as “0” as not all effect sizes were >2 or <0.5 and statistically significant.

The final overall GRADE Score [26] was very low (score of one or less).

### Results of individual studies

A Forest plot was prepared for effect estimates and confidence intervals for anti-psychotics as a class (Figure 1) and of each drug (risperidone, quetiapine, olanzapine and aripiprazole in Figures 2, 3, 4, 5). One study evaluated paliperidone, which is the active metabolite of risperidone, and was therefore included with the trials of risperidone.

### Synthesis of results

Fourteen studies with 493 participants (242 atypical antipsychotic and 251 placebo) were identified. The overall mean difference in Y-BOCS score change between drug and placebo groups was 2.34 points which had an overall effect-size of *D* = 0.40 (Figure 1). This is equivalent to about 10% reduction in Y-BOCS for those taking antipsychotics score over time.

**Figure 8:**
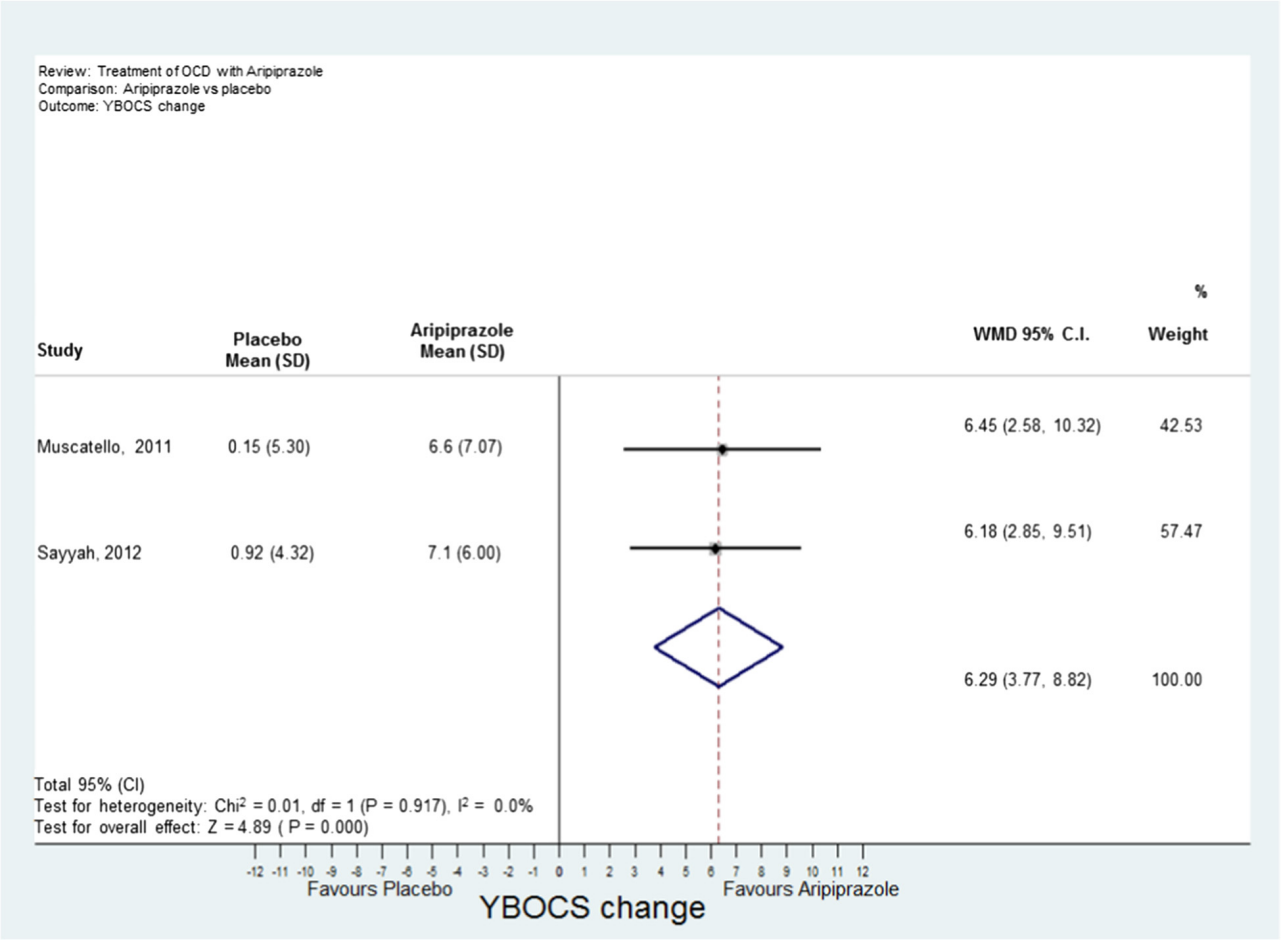
**Meta-analysis of risperidone treatment vs placebo for obsessive-compulsive disorder, measured as odds ratios.**
- (b) Olanzapine: Two studies were identified [13,18] with 35 participants taking olanzapine and 35 taking a placebo. The overall difference between olanzapine and placebo was −0.19, less than one unit point on the Y-BOCS. This difference was non-significant (Figure 3).
- (c) Quetiapine: Five studies were identified [19–23] with 89 participants taking quetiapine and 89 placebo. The overall difference between quetiapine and placebo was not significant (0.81 Y-BOCS units) (Figure 4).
- (d) Aripiprazole: Two studies were identified [24,25] with 41 participants taking aripiprazole and 38 taking placebo. The overall difference between aripiprazole and placebo was statistically and clinically significant with a difference in Y-BOCS outcome scores of 6.29 units and overall effect size of *D* = 1.11 (Figure 5).

- The results of the individual atypical anti-psychotics were as follows:
- (a) Risperidone: Five studies were identified [12,14–17] with 77 participants in total taking risperidone and 89 receiving placebo. The overall difference was statistically significant with an overall mean reduction of 3.89 points on the Y-BOCS (95% CI = 1.43-5.48) and an effect size of *D* = 0.53 (Figure 2). The categorical analyses of responders in comparison to non-responders, on the Y-BOCS, indicated that overall those participants taking risperidone were 3.10 times more likely to respond to treatment (see Figure 8). The number needed to treat (NNT) for this ratio was 4.65.

### Narrative review

- (a) Dose
- Within risperidone trials, one study [15] used a very low fixed dose of risperidone (0.5 mg) and had a better effect-size than all the studies that used a moderate dose. This pattern was not possible to identify with quetiapine studies which used a low to moderate dose range. Kordon [22] used the highest dose of quetiapine out of the all quetiapine studies and there was no significant benefit.
- (b) Duration of antipsychotic trial
- A variety of end-points were used from 6 weeks to 16 weeks. Six studies [12,13,16,19,20,23] repeated the Y-BOCS every 1 to 4 weeks before their end-point. There was no discernible pattern of effect-size on the length of the trial. Duration of 4 weeks or more did not seem to make any difference to response. However one study [23] found that the quetiapine group became significantly worse between week 4 and week 12.
- (c) Duration of SSRI prior to trial
- Four studies [12,13,15,23] were preceded by an open-label study of a SSRI to determine responsiveness prior to commencing the anti-psychotic trial. The remainder studies recruited patients who were on a SSRI as part of their routine care where it may be more difficult to judge the treatment resistant criteria.
- Most studies recruited participants who had been on a SSRI for 12 weeks. However three studies [13,19,24] included participants who had been on a SSRI for only 8 weeks. Of these, only one [19] had any significant benefit from augmentation. A short duration of SSRI used may be a source of bias in a small study as OCD may respond to SSRIs gradually with some patients responding more slowly than others. Of these three studies, Erzegovesi [15] also investigated effect of augmenting SRRI *responders* with risperidone and found no difference between risperidone and placebo.
- (d) Previous CBT
- Previous CBT is an integral part of stepped care in the NICE guidelines. Only four out of the 14 studies [14,18,19,22] recruited a majority (range 62.5-100%) of participants who had had a previous trial of CBT. Of these, two of the four studies found significant benefit. None of the studies had a trial of CBT as one of their treatment resistant criteria or had any formal assessment of the adequacy of such trials.
- (e) Treatment refractoriness and SSRI treatment resistant criteria
- There was no discernible pattern in effect-size for the degree of pharmacological treatment refractoriness (e.g. number of SSRIs) or stringency of SSRI resistant criteria. All of the studies recruited subjects with a Y-BOCS of moderate severity in the range of 20 – 30. Severe symptoms of OCD begin with a Y-BOCS >30. Studies reporting a higher Y-BOCS scores before randomization had a larger effect-size for risperidone [12,14,15] or quetiapine [19] than studies with a lower Y-BOCS which suggests regression to the mean. However there were no baseline differences between the groups in these studies.
- (f) Additional treatment arms
- Two studies had an additional treatment arm. Simpson et al., [16] evaluated CBT as an additional arm and found that adding CBT was superior to adding either risperidone or placebo. Diniz et al. [23] found that adding clomipramine (25-75 mg) to fluoxetine or adding a placebo to fluoxetine was superior to quetiapine use. However in this study, for participants taking 60-80 mg, the dose of fluoxetine was reduced to avoid interaction with clomipramine.
- (g) Follow up
- None of the studies had any long-term follow-up for outcome or adverse events after their end point. One study [33] (which was excluded from the meta-analysis as it was a follow up study with a variety of anti-psychotics) compared participants who had responded to a SSRI plus CBT for 1 year. Subjects who failed to respond to a SSRI were randomly assigned to quetiapine, risperidone or olanzapine plus CBT. At 1-year follow-up, augmentation with CBT and an antipsychotic was associated with a drop of 10 points on the Y-BOCS. However their Y-BOCS remained significantly higher compared to the SSRI responders after 1 year and both groups had received CBT. Fifty per-cent of subjects on the antipsychotic had an increase of >10% in their Body Mass Index (BMI) and a higher fasting blood sugar compared to 15.2% with raised BMI in the SSRI responders.
- (h) Differences in pharmacodynamics
- The anti-psychotic, haloperidol (which is highly selective for D2 receptors) was shown to be effective against a placebo in one early study [9], which achieved Y-BOCS change of 5 units compared to placebo with an effect size of *D* =1.06. Aripiprazole is the most atypical (in terms of effects on D2, 5HT-1A and 2A, and 5HT-C receptors) and also showed a similar effect size of 6 units over placebo. Thus there dos not appear to any specific pharmacodynamics effect of anti-psychotics in OCD and that the differences between studies are more likely to occur because of the heterogeneity within OCD.
- (i) Non-Responders by symptom sub-type
- No studies specifically report excluding hoarders, which is now recognized as a separate disorder in DSM-5 and generally has a worse prognosis with any treatment. Two studies [12,19] attempted to classify their participants according to predominant symptom subtype (for example the dimensions of checking; symmetry, order, counting and repeating; contamination and cleaning; hoarding) [34]. Certain symptom sub-types might do better or worse with a treatment although sub-types often overlap and vary in severity. As yet there is no identified genotype or phenotype to determine predictors of outcome with an anti-psychotic augmentation in OCD.
- (j) Tic disorder
- The original study on haloperidol [12] found benefit in those with comorbid tics compared to those without. In this meta-analysis, two studies found no difference in response between those with or without co-morbid tics [12,20]. In all other studies, no analyses were made of co-morbid tics either because of the small numbers or no assessment was made.

## Discussion

This systematic review and meta-analysis found evidence for the benefit with a modest effect size for aripiprazole in the short term in people with OCD who were resistant to at least one SSRI. Risperidone or anti-psychotics as a class had a statistically significant benefit in the short term but with a weaker effect size. There was no evidence for the clinical effectiveness of olanzapine or quetiapine. The overall GRADE of the recommendations of anti-psychotics in OCD was very low [26].

The strength of this study is that it is an up to date systematic review and meta-analysis of antipsychotic augmentation in OCD since the publication of five recent RCTs. It was conducted using a thorough search of publicly accessible databases, and by requesting unpublished studies from pharmaceutical companies. A number of possible biases were identified that may result in an over-estimate of treatment benefits or difficulties in generalizing to the population of treatment refractory OCD.

The heterogeneity and weakness of the effect size of anti-psychotics as a class may be because of the heterogeneous nature of OCD, different populations recruited and small sample sizes. Thus it may be that there is a sub-group of people with OCD who may respond to any anti-psychotic as a class effect – the problem is at present there is no way of identifying “responders” before a trial.

Although we found benefit for aripiprazole and risperidone, this should be weighed against unknown benefit and potential physical risks in the long term. Aripiprazole is limited to two recent studies. Thus it would be particularly important to conduct large studies of aripiprazole in participants who have failed at least one SSRI and CBT to determine effectiveness in the long term. However it may not be particularly helpful to conduct further trials of other anti-psychotics in OCD until bio-psychosocial markers can identify the minority who may respond. Furthermore it would be helpful if researchers could agree to treatment resistant criteria before entry into such a trial. Psychopharmacologists prefer different percentage changes on the Y-BOCS [35] whilst cognitive behaviour therapists tend to use the Jacobson & Truax [36] index of "reliable and significant change" and to complement the YBOCS with subject rated symptom and quality of life measures.

Alternative augmentation strategies may be more effective than an antipsychotic and safer in the long term. For example, from a study in this meta-analysis, Simpson et al., [16] found that adding CBT was superior to adding either risperidone or placebo. Diniz et al., [23] found that adding clomipramine 25-75 mg to fluoxetine was superior to quetiapine and fluoxetine. Another randomised open-label trial found that citalopram with clomipramine was superior to citalopram alone [37]. These studies have possible implications for recommending the order of treatments in the stepped care of OCD: for adults with OCD on a SSRI there is an argument for a more intensive trial of CBT before a trial of anti-psychotic. Another alternative is combining citalopram or escitalopram with a low dose of clomipramine with ECG monitoring of the QTc interval. Caution would be required combining fluoxetine with clomipramine because of interactions on the hepatic cytochrome P450 iso-enzymes. This can potentially lead to an increase of clomipramine so that plasma clomipramine levels and ECG monitoring would be required. It is not known whether combining a SSRI with clomipramine would be equally effective as increasing the SSRI to a supra-maximal dose with serum level and ECG monitoring [38].

The area in which there is least knowledge for treatment algorithms is for people with OCD with severe symptoms who have been resistant to at least 2 trials of SSRI or clomipramine for a minimum of 12 weeks at maximum tolerated dose and two trials of CBT, which has been competently delivered. None of the current studies had a trial of even one CBT as one of their treatment resistant criteria for entry into their trial or had any formal assessment of the adequacy of such trials. A combination of SSRI and CBT can augment the effect-size in severe OCD. However it is not known if a SSRI with an antipsychotic can augment or even diminish the effect of CBT.

The clinical implications are that if aripiprazole or risperidone is used in severe treatment resistant OCD, then to determine effectiveness it should be a trialled for no longer than 4 weeks and without any other interventions such as CBT to determine effectiveness. Erzegovesi [15] also found that there was no difference to augmenting SRRI *responders* with risperidone and placebo. Thus for patients who have had a response to a SSRI (but are usually still symptomatic), they may not obtain any extra benefit from adding risperidone.

For risperidone a low dose of 0.5 mg or for aripiprazole 10 mg (or possibly lower) may be recommended in those who have not responded to two trials of SSRI or CBT. If a patient is judged to be a responder at 4 weeks then a full discussion should be had with the patient on the possible long-term adverse risks and the need for regular monitoring of weight, blood sugar and lipid profile. Audits of referrals of patients with OCD on anti-psychotics at our specialist service suggest that outcome monitoring at 4 weeks or physical monitoring in the long term are rarely conducted.

## Conclusions

In summary, we found limited evidence for low dose risperidone and aripiprazole in the short-term. Aripiprazole is associated with less risk of weight gain, sedation, and increase in prolactin compared to other antipsychotics [10]. We do not recommend the use of olanzapine or quetiapine to augment SSRIs in OCD. There is some evidence for augmenting a SSRI with CBT or clomipramine before an anti-psychotic. However a combination with clomipramine requires ECG monitoring. The definition of treatment resistance should include at least one adequate trial of CBT. Studies of augmentation of a SSRI with aripiprazole should be followed up in the long-term.
